# Assessment of antibiotic resistance changes during the Covid-19 pandemic in northeast of Iran during 2020–2022: an epidemiological study

**DOI:** 10.1186/s13756-022-01159-y

**Published:** 2022-10-01

**Authors:** Reza Khoshbakht, Mona Kabiri, Alireza Neshani, Mohammad Navid Khaksari, Sayyed Majid Sadrzadeh, Seyed Mohammad Mousavi, Kiarash Ghazvini, Mahdis Ghavidel

**Affiliations:** 1 Department of Laboratory Sciences, School of Paramedical Sciences, Mashhad University of Medical Sciences, Mashhad, Iran; 2 Student Research Committee, Mashhad University of Medical Sciences, Mashhad, Iran; 3 Clinical Research Development Unit, Ghaem Hospital, Faculty of Medicine, Mashhad University of Medical Sciences, Mashhad, Iran; 4 Department of Emergency Medicine, Faculty of Medicine, Mashhad University of Medical Sciences, Mashhad, Iran; 5 Department of Microbiology and Virology, School of Medicine, Mashhad University of Medical Sciences, Mashhad, Iran; 6 Antimicrobial Resistance Research Center, Mashhad University of Medical Sciences, Mashhad, Iran; 7 Shahid Hasheminejad Hospital, Mashhad University of Medical Sciences, Mashhad, Iran

**Keywords:** Antibiotic resistance, COVID-19 pandemic, *Escherichia coli*, *Pseudomonas aeruginosa*, *Klebsiella pneumoniae*, *Acinetobacter baumannii*

## Abstract

**Background:**

The coronavirus disease 2019 seems to change antibiotic resistance pattern. Certain conditions in the Covid-19 era may be contributing to the rise of antimicrobial resistance (AMR). Due to the limited information on the impact of Covid-19 on antimicrobial resistance (AMR), the purpose of this research was to investigate the trend in antimicrobial resistance changes of *E. coli, P. aeruginosa, K. pneumoniae,* and *A. baumannii* in Hasheminezhad hospital. This hospital was a Corona center in Mashhad at the onset of this epidemic.

**Methods:**

1672 clinical samples were collected between January 21, 2020 and January 30, 2022from patients hospitalized at Hasheminezhad Hospital in Mashhad, Conventional microbiological procedures for identifying gram-negative bacteria and antibiotic susceptibility testing were used, according to the clinical and laboratory standards institute (CLSI) 2021. The two years of the pandemic, from the initial stage of the outbreak until the 6th peak, (January 2020 to and January 2022) were divided into 9 periods according to the seasons.

**Results:**

Highest resistance rates were seen in *E. coli* (615 samples)*, K. pneumoniae* (351 samples)*, P. aeruginosa* (362 samples) and *A. baumannii* (344 samples) to Ampicillin (89.6%), Ampicillin (98%), Imipenem (91.8%), and Ceftazidime (94.6%), respectively. The largest change in antibiotic resistance was seen between Summer 2020 and Summer 2021 for *K. pneumoniae* with about a 30% rise in antibiotic resistance to Ceftriaxone.

**Conclusions:**

All 4 species evaluated in this study, have shown rising AMR rates during the first year of the pandemic in the northeast of Iran. This study revealed that *E. coli, P. aeruginosa, K. pneumoniae,* and *A. baumannii* strains in Northern Iran have a higher level of antibiotic resistance than what was measured in similar studies conducted before the pandemic. This will further restrict treatment choices and jeopardize global public health.

## Background

The coronavirus disease 2019 (Covid-19) is increasingly spreading over the world. Covid-19 is known to cause severe pneumonia, as well as acute respiratory distress syndrome (ARDS) and significant mortality rates. Despite enormous attempts to manage the pandemic, the number of people infected and mortality continue to rise. Covid-19 has been linked to about 500 million cases and over 6 million deaths as of Apr 7, 2022 [1]. The Covid-19 pandemic seems to have a significant influence on public health, influencing the management of a variety of health care issues, including antimicrobial resistance (AMR). Several studies have found a relationship between Covid-19 and AMR, suggesting that some conditions, often including increased antibiotic usage, may be contributing to the rise of AMR [2]. Although antibiotics were used in 72 percent of Covid-19 patients, only 8 percent of hospitalized Covid-19 patients were found to suffered from a fungal or bacterial infection [3]. While experts have tried to warn of a relationship between AMR and Covid-19, research has shown conflicting results. In the Covid-19 pandemic, several studies have found outbreaks or an increase in diseases caused by multidrug-resistant bacteria. However, according to other research, the number of infections caused by multidrug-resistant bacteria has not increased [4].

According to the WHO, AMR is among the top ten worldwide health threats, and while it receives less attention than Covid-19, it may have just as severe negative outcomes. In 2017, the WHO named a series of bacteria of specific concern for which novel antibiotics are needed. *Escherichia coli*, *Pseudomonas aeruginosa*, *Klebsiella pneumoniae*, and *Acinetobacter baumannii* are among the critical bacteria that cause the most harm to humans health and should be prioritized in the development of novel antimicrobial therapies [5].

*P. aeruginosa* is the most frequent gram-negative bacteria that cause ventilator-associated pneumonia and the second most prevalent organism responsible for catheter-associated urinary tract infections (UTIs). This bacterium is resistant to many antibiotics. *K. pneumoniae* is the most common gram-negative bacteria causing central line-associated bloodstream infections and is one of the primary drivers of AMR nosocomial infections globally.

*E. coli* is the most prevalent pathogen responsible for UTIs and the second most frequent pathogen responsible for healthcare-associated infections. During the past several decades, a growing number of resistance genes have been found in *E. coli* isolates, many of which have been acquired by horizontal gene transfer [6, 7]. *A. baumannii* is the most common pathogen causing infection in hospitals and is now regarded as a global problem in the healthcare system due to its proclivity to acquire multidrug-resistant features at previously unanticipated rates [8]*.* However, there is currently a lack of information regarding the effects of COVID-19 on AMR. COVID-19 and AMR are two considerable health threats. Nevertheless, there are limited data on the relationship between them. In addition, the gram-negative bacteria mentioned in clinical settings are highly important. Consequently, the present study aimed to evaluate the AMR of *E. coli*, *P. aeruginosa*, *K. pneumoniae*, and *A. baumannii* at Hasheminezhad Hospital during the two-year COVID-19 pandemic. Moreover, we compared the antibiotic resistance pattern between nine outbreak seasons.

## Method

### Patients and samples

This cross-sectional study was conducted between January 2020 (21 Jan) and January 2022 (30 Jan) in Mashhad, Hasheminezhad Hospital. This hospital has become a Corona center after pandemic. Overall, 1672 patients were referred to the laboratory. During the study period, 1672 clinical samples were collected, including urine, blood, respiratory secretions, wounds, and other specimens. All samples were sent to the central laboratory and because of cross resistance phenomenon, multi samples from an individual or samples from patients with polymicrobial infection have been excluded from study.

### Samples identification

The samples were processed using standard microbiological procedures for identifying gram-negative bacteria. Standard biochemical procedures and protocols were utilized to recognize and identify the strains of *Escherichia coli*, *Pseudomonas aeruginosa*, *Klebsiella pneumoniae*, and *Acinetobacter baumannii*. The samples were cultured on MacConkey agar (Merck) medium and blood agar (Merck) and incubated at room temperature for 24 h. To identify the strains, routine biochemical tests such as urea urease, oxidase, citrate, triple sugar iron agar (TSI), malonate consumption, sugar oxidation and fermentation, Methyl Red motility, Voges-Proskauer, and indole production were used.

### Antimicrobial susceptibility test

Ampicillin (AP), Ampicillin-Sulbactam (SAM), Amikacin (AK), Piperacillin-Tazobactam (PTZ), Cefazolin (CZ), Cefepime (CPM), Ceftriaxone (CRO), Cefoxitin (FOX), Ceftazidime (CAZ), Imipenem (IMI), Meropenem (MEM), Gentamicin (GM), Co-trimoxazole (TS), Nitrofurantoin (NI), Nalidixic acid (NA), Ciprofloxacin (CIP), Cefotaxime (CTX), Levofloxacin (LEV), Cefixime (CFM), and Cefoperazone (CP) were the antibacterial drugs contained in the panel isolates. A specific antibiogram panel considered for each bacterium was based on the type of bacteria according to the clinical and laboratory standards institute (CLSI) 2021 guidelines [9].

### Statistical analysis

Data were analyzed using SPSS software, version 22. The frequency (percentage) was used to describe the qualitative variables. The age variable was checked by the Kolmogorov–Smirnov test for normality and the mean ± standard deviation (SD) was reported. The Chi-square or Fisher exact test was used to compare the qualitative variables. The antibiotic resistance pattern for each antibiotic has been compared in various seasons. January 2020 to January 2022 is separated into 9 periods based on seasons. Winter 2020, spring 2020, summer 2020, fall 2020, winter 2021, spring 2021, summer 2021, fall 2021, and winter 2022 are the seasons considered in the present study. A significant level of *P* < 0.05 was considered statistically significant with %95 confidence interval (CI).

## Results

### Bacterial isolates

In this study, 1672 isolates were collected from 781 (46.7%) females, 843 (50.4%) males, and 48 (2.9%) of unknown gender admitted at Hasheminezhad hospital. Among 1672 bacterial isolates, 615 (36.8%) were *E. coli*, 362 (21.6%) isolates were *P. aeruginosa*, 351 (20.9%) were *K. pneumoniae*, and 344 (20.5%) isolates were *A. baumannii*. The mean age of patients was 54.77 ± 24.16. The frequency of isolates based on sample type and hospital wards has been shown in Tables 1 and 2.

**Table 1:** Sample type distribution of 1672 isolates

Sample	Frequency	Percentage
Urine	973	58.2
Trachea	438	26.2
Wound	74	4.4
Blood	42	2.5
Lung	37	2.2
Throat	32	1.9
Unknown	24	1.4
Eye	10	0.6
Catheter	7	0.4
Ascites fluid	7	0.4
Pleural fluid	7	0.4
Tracheostomy	7	0.4
CSF	4	0.2
Abscess	2	0.1
Sputum	2	0.1
Joint fluid	2	0.1
Other fluids	2	0.1
Peritoneum	1	0.1
Bile fluid	1	0.1

**Table 2:** Frequency distribution of 1672 isolates based on hospital wards

Ward	Frequency	Percentage
ICU	844	50.5
Unknown	242	14.5
Out-patient	114	6.8
Emergency	113	6.8
Respiratory	73	4.4
Heart	71	4.2
Pediatrics	43	2.6
Surgery	43	2.6
Internal	38	2.3
Infectious	26	1.6
NICU	21	1.3
CCU	13	0.8
Midwifery	12	0.7
Poisoning	12	0.7
Orthopedics	4	0.2
Neurology	3	0.2

### Antibiogram results by agar disc diffusion method

The antibiotic resistance patterns of all isolates are presented in Table 3. For *E. coli*, the highest resistance was to Ampicillin (89.6%), Cefazolin (74.0%), and Cefepime (71.4%). For *K. pneumoniae* the highest resistance was to Ampicillin (98.1%), Levofloxacin (92.9%), and Ceftazidime (92.4%). For *P. aeruginosa* the highest resistance was to Imipenem (91.8%), Meropenem (91.5%), and Cefepime (87.1%). Finally, for *A. baumannii* the highest resistance was to Cefotaxime (94.7%), Ceftazidime (94.6%), and Ceftriaxone (93.4%).

**Table 3:** Antibiotic resistance patterns of *E. coli, K. pneumoniae, P. aeruginosa,* and *A. baumannii*

Species (number of isolates)	Antibiotic resistance patterns	AP	SAM	PTZ	CZ	CPM	CRO	FOX	IMI	MEM	GM	AK	CIP	NI	LEV	CTX	CAZ
*E. coli (615)*	Resistant	69 (89.6%)	56 (58.9%)	22 (36.7%)	313 (74.0%)	60 (71.4%)	393 (67.6%)	30 (47.6%)	222 (42.7%)	154 (33.6%)	88 (17.7%)	19 (31.7%)	15 (45.5%)	96 (20.2%)	–	–	33 (70.2%)
	Intermediate	1 (1.3)	13 (13.7%)	8 (13.3%)	1 (0.2%)	8 (9.5%)	22 (3.8%)	5 (7.9%)	132 (25.4%)	78 (17.0%)	17 (3.4%)	6 (10.0%)	8 (24.2%)	67 (14.1%)	–	–	3 (6.4%)
	Susceptible	7 (9.1%)	26 (27.4%)	30 (50.0%)	109 (25.8%)	16 (19.0%)	166 (28.6%)	28 (44.4%)	166 (31.9%)	227 (49.5%)	391 (78.8%)	35 (58.3%)	10 (30.3%)	313 (65.8%)	–	–	11 (23.4%)
*K. pneumoniae*	Resistant	101 (98.1%)	132 (81.0%)	81 (72.3%)	161 (80.5%)	122 (86.5%)	255 (78.9%)	80 (70.8%)	213 (71.7%)	197 (69.4%)	98 (38.3%)	50 (53.2%)	12 (70.6%)	93 (72.7%)	13 (92.9%)	13 (76.5%)	73 (92.4%)
(351)	Intermediate	0 (0.0%)	13 (8.0%)	17 (15.2%)	2 (1.0%)	10 (7.1%)	11 (3.4%)	7 (6.2%)	37 (12.5%)	29 (10.2%)	10 (3.9%)	7 (7.4%)	2 (11.8%)	9 (7.0%)	0 (0.0%)	0 (0.0%)	1 (1.3%)
	Susceptible	2 (1.9%)	18 (11%)	14 (12.5%)	37 (18.5%)	9 (6.4%)	57 (17.6%)	26 (23.0%)	47 (15.8%)	58 (20.4%)	148 (57.8%)	37 (39.4%)	3 (17.6%)	26 (20.3%)	1 (7.1%)	4 (23.5%)	5 (6.3%)
*P. aeruginosa*	Resistant	–	–	216 (79.1%)	–	243 (87.1%)	–	–	290 (91.8%)	303 (91.5%)	220 (70.5%)	239 (79.4%)	13 (61.9%)	–	240 (85.7%)	–	252 (86.3%)
(363)	Intermediate	–	–	18 (6.6%)	–	8 (2.9%)	–	–	5 (1.6%)	1 (0.3%)	9 (2.9%)	9 (3.0%)	4 (19.0%)	–	16 (5.7%)	–	10 (3.4%)
	Susceptible	–	–	39 (14.3%)	–	28 (10.0%)	–	–	21 (6.6%)	27 (8.2%)	83 (26.6%)	53 (17.6%)	4 (19.0%)	–	24 (8.6%)	–	30 (10.3%)
*A. baumannii*	Resistant	–	187 (77.9%)	199 (89.6%)	–	155 (89.6%)	241 (93.4%)	–	206 (88.4%)	248 (88.9%)	139 (70.9%)	151 (73.3%)	3 (42.9%)	–	198 (88.0%)	233 (94.7%)	226 (94.6%)
(344)	Intermediate	–	24 (10.0%)	12 (5.4%)	–	5 (2.9%)	8 (3.1%)	–	6 (2.6%)	3 (1.1%)	4 (2.0%)	4 (1.9%)	2 (28.6%)	–	6 (2.7%)	9 (3.7%)	2 (0.8%)
	Susceptible	–	29 (12.1)	11 (5.0%)	–	13 (7.5%)	9 (3.5%)	–	21 (9.0%)	28 (10.0%)	53 (27.0%)	51 (24.8%)	2 (28.6%)	–	21 (9.3%)	4 (1.6%)	11 (4.6%)

AP (Ampicillin), SAM (Ampicillin-Sulbactam), PTZ (Pipracillin-Tazobactam), CZ (Cefazolin), CPM (Cefepime), CRO (Ceftriaxone), FOX (Cefoxitin), IMI (Imipenem), MEM (Meropenem), GM (Gentamicin), AK (Amikacin), CIP (Ciprofloxacin), NI (Nitrofurantoin), LEV (Levofloxacin), CTX (Cefotaxime), and CAZ (Ceftazidim)

*Samples for which antibiotic resistance testing has not been performed are marked with "–"

* Percentage of each column is calculated based on the sum of 3 rows.

## Results by season

**Fig. 1:**
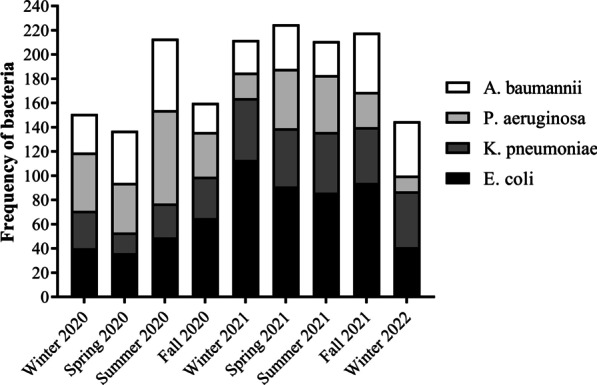
Frequency of samples sent to the laboratory-based on seasons

The number of samples sent to the laboratory according to the seasons is shown in Fig. 1. Seasons with the highest number of samples in *E. coli, P. aeruginosa, K. pneumoniae,* and *A. baumannii* were Winter 2021 (113), Winter 2021 (51), Summer 2020 (77), and Summer 2020 (59) respectively (*P* < 0.001).

Antibiotic resistance patterns of 9 seasons of pandemic between January 2020 and January 2022 have been shown in Tables 4, 5, 6, and 7. Antibiotics selected for each bacterium were based on CLSI 2021. There was no significant difference in Ceftriaxone, Levofloxacin, and Nitrofurantoin for *K. pneumoniae* and Ciprofloxacin for *A. baumannii*. For *E. coli*, the most change in antibiotic resistance in Cefazolin was between Fall 2020 (58.6%) and Summer 2021 (85.9%). For *K. pneumoniae*, the most change in antibiotic resistance in Imipenem was between Spring 2020 (20%) and Summer 2021 (83.7%). For *P. aeruginosa*, the most change in antibiotic resistance in Meropenem was between Summer 2020 (82.8%) and Spring 2021 (100%). For *A. baumannii*, the most change in antibiotic resistance in Cefepime was between Winter 2020 (80%) and Summer 2021 (100%).

**Table 4:** Antibiotic resistance pattern in *E. coli* based on seasons

Antibiotic	Season									
	Winter 2020	Spring 2020	Summer 2020	Fall 2020	Winter 2021	Spring 2021	Summer 2021	Fall 2021	Winter 2022	*P* value
AP	–	5 (83.3)	6 (66.6)	4 (80.0)	23 (95.8)	20 (95.2)	5 (100)	2 (66.6)	4 (100)	< 0.001
CZ	3 (100)	22 (66.6)	35(72.9)	34 (58.6)	42 (67.7)	47 (87.0)	61 (85.9)	52 (71.2)	17 (80.9)	< 0.001
GM	7 (63.6)	11 (31.4)	9 (18.7)	10 (16.3)	17 (16.1)	10 (12.3)	7 (11.6)	15 (15.6)	2 (33.3)	< 0.001
AK	2 (100)	0 (0)	4 (44.4)	2 (50)	4 (66.6)	4 (25)	2 (28.5)	1 (10)	–	< 0.001
SAM	–	2 (33.3)	1(14.2)	4 (80)	15 (71.4)	9 (60)	8 (47)	12 (70.5)	5 (71.4)	0.002
PTZ	–	0 (0)	1 (14.2)	0 (0)	13 (59)	0 (0)	0 (0)	6 (50)	2 (33.3)	< 0.001
CPM	–	1 (25)	6 (60)	2 (40)	16 (80)	14 (82.3)	10 (62.5)	11 (91.6)	–	0.005
FOX	1 (33.3)	2 (33.3)	1 (16.6)	1 (20)	11 (73.3)	8 (61.5)	–	2 (66.6)	4 (33.3)	< 0.001
CRO	22 (61.1)	18 (52.9)	30 (69.7)	32 (52.4)	70 (67.3)	68 (76.4)	65 (76.4)	64(68)	24 (68.5)	< 0.001
CIP	–	–	–	9 (56.2)	3 (50)	2 (25)	0 (0)	1 (100)	–	< 0.001
IMI	1 (11.1)	0 (0)	12 (25)	25 (40.9)	45 (42.8)	59 (85.5)	44 (61.1)	24 (28.9)	12 (32.4)	< 0.001
MEM	1 (9)	0 (0)	4 (8.3)	3 (4.6)	64 (68.8)	56 (70.8)	11 (36.6)	12 (13.3)	3 (27.2)	< 0.001
CAZ	–	4 (57.1)	6 (75)	4 (80)	4 (50)	1 (50)	2 (66.6)	3 (75)	9 (90)	< 0.001
NI	3 (9.3)	12 (46.1)	20 (54)	15 (25.4)	7 (8.4)	8 (11.1)	19 (28.7)	11 (14.8)	1 (3.7)	< 0.001

AP (Ampicillin), CZ (Cefazolin), GM (Gentamicin), AK (Amikacin), SAM (Ampicillin-Sulbactam), PTZ (Pipracillin-Tazobactam), CPM (Cefepime), FOX (Cefoxitin), CRO (Ceftriaxone), CIP (Ciprofloxacin), IMI (Imipenem), MEM (Meropenem), CAZ (Ceftazidim) and NI (Nitrofurantoin).

Variables were described as the frequency (%).

*Samples for which antibiotic resistance testing has not been performed are marked with "–".

**Table 5:** Antibiotic resistance pattern in *K. pneumoniae* based on seasons

Antibiotic	Season									
	Winter 2020	Spring 2020	Summer 2020	Fall 2020	Winter 2021	Spring 2021	Summer 2021	Fall 2021	Winter 2022	*P* value
AP	2 (66.6)	7 (100)	15 (100)	13 (92.8)	28 (100)	22 (100)	6 (100)	3 (100)	5 (100)	< 0.001
CZ	1 (50)	11 (78.5)	15 (68.1)	10 (58.2)	24 (80)	25 (86.2)	41 (93.1)	23 (82.1)	11 (78.5)	< 0.001
GM	12 (63.1)	7 (41.1)	9 (34.6)	16 (51.6)	23 (47.9)	11(31.4)	5 (13.5)	14 (33.3)	1 (100)	< 0.001
AK	3 (75)	2 (28.5)	9 (56.2)	13 (68.4)	3 (75)	10 (25)	4 (57.1)	6 (54.5)	–	< 0.001
SAM	–	2 (33.3)	11 (91.6)	12 (100)	24 (82.7)	22 (84.6)	21 (65.6)	24 (88.8)	16 (84.2)	< 0.001
PTZ	3 (75)	4 (57.1)	12 (75)	13 (68.4)	23 (79.3)	3 (100)	0 (0)	18 (75)	5 (62.5)	< 0.001
CPM	2 (100)	6 (75)	12 (75)	14 (82.3)	25 (92.5)	20 (86.9)	25 (89.2)	17 (89.4)	1 (100)	< 0.001
FOX	2 (100)	4 (57.1)	10 (83.3)	10 (66.6)	21 (77.7)	11 (61.1)	0 (0)	2 (100)	20 (68.9)	< 0.001
CTX	6 (85.7)	–	1 (50)	3 (100)	–	2 (100)	0 (0)	1 (50)	–	0.001
CRO	22 (84.6)	12 (75)	16 (61.5)	22 (64.7)	40 (83.3)	39 (82.9)	43 (91.4)	31 (77.5)	30 (76.9)	0.070
CIP	–	–	–	5 (71.4)	2 (100)	0 (0)	4 (80)	1 (100)	–	0.007
LEV	2 (100)	–	–	3 (100)	–	2 (100)	1 (100)	4 (80)	1 (100)	0.219
IMI	7 (38.8)	3 (20)	17 (65.3)	23 (69.6)	37 (75.5)	31 (81.5)	36 (83.7)	30 (83.3)	29 (74.3)	< 0.001
MEM	11 (55)	5 (31.2)	13 (48.1)	18 (54.5)	45 (91.8)	39 (84.7)	22 (66.6)	32 (69.5)	12 (85.7)	< 0.001
CAZ	6 (100)	8 (88.8)	13 (86.6)	12 (100)	1 (100)	1 (100)	2 (100)	8 (100)	22 (88)	< 0.001
NI	3 (9.3)	12 (46.1)	20 (54)	15 (25.4)	7 (8.4)	8 (11.1)	19 (28.7)	11 (14.8)	1 (3.7)	0.472

AP (Ampicillin), CZ (Cefazolin), GM (Gentamicin), AK (Amikacin), SAM (Ampicillin-Sulbactam), PTZ (Pipracillin-Tazobactam), CPM (Cefepime), FOX (Cefoxitin), CTX (Cefotaxime), CRO (Ceftriaxone), CIP (Ciprofloxacin), IMI (Imipenem), MEM (Meropenem), CAZ (Ceftazidim) and NI (Nitrofurantoin).

Variables were described as the frequency (%).

*Samples for which antibiotic resistance testing has not been performed are marked with "–".

**Table 6:** Antibiotic resistance pattern in *P. aeruginosa* based on seasons

Antibiotic	Season									
	Winter 2020	Spring 2020	Summer 2020	Fall 2020	Winter 2021	Spring 2021	Summer 2021	Fall 2021	Winter 2022	*P* value
CAZ	21 (95.4)	34 (85)	63 (86.3)	35 (100)	4 (66.6)	29 (93.5)	38 (82.6)	19 (76)	8 (61.5)	< 0.001
GM	28 (93.3)	33 (86.4)	62 (82.6)	30 (83.3)	8 (40)	31 (72)	14 (32.5)	13 (50)	–	< 0.001
PTZ	17 (89.4)	25 (65.7)	61 (80.2)	34 (94.4)	13 (61.9)	31(88.5)	6 (75)	19 (73)	9 (69.2)	< 0.001
AK	18 (94.7)	27 (81.8)	59 (84.2)	30 (88.2)	11 (61.1)	34 (77.2)	36 (78.2)	18 (64.2)	5 (62.5)	< 0.001
CPM	10 (90.9)	32 (96.9)	67 (88.1)	32 (91.4)	9 (56.2)	32 (91.4)	38 (88.3)	13 (68.4)	9 (75)	< 0.001
CIP	–	0 (0)	–	9 (90)	2 (40)	–	0 (0)	–	1 (100)	< 0.001
LEV	15 (93.7)	33 (91.6)	62 (84.9)	33 (94.2)	12 (66.6)	35 (89.7)	17 (73.9)	23 (82.1)	9 (81.8)	< 0.001
IMI	26 (89.6)	31 (81.5)	67 (89.3)	34 (97.1)	16 (80)	32 (96.9)	42 (95.4)	29 (100)	12 (100)	< 0.001
MEM	25 (89.2)	34 (85)	63 (82.8)	35 (97.2)	16 (84.2)	48 (100)	46 (97.8)	28 (96.5)	7 (100)	< 0.001

CAZ, Ceftazidime; GM, Gentamicin; PTZ, Piperacillin-Tazobactam; AK, Amikacin; CPM, Cefepime; CIP, Ciprofloxacin; LEV, Levofloxacin; IMI, Imipenem; MEM, Meropenem

Variables were described as the frequency (%)

*Samples for which antibiotic resistance testing has not been performed are marked with "–"

**Table 7:** Antibiotic resistance pattern in *A. baumannii* based on seasons

Antibiotic	Season									
	Winter 2020	Spring 2020	Summer 2020	Fall 2020	Winter 2021	Spring 2021	Summer 2021	Fall 2021	Winter 2022	*P* value
SAM	2 (100)	16 (48.4)	32 (82)	9 (90)	17 (100)	22 (75.8)	21 (95.4)	33 (73.3)	35 (81.3)	< 0.001
CAZ	6 (66.6)	32 (86.4)	31 (93.9)	18 (100)	5 (100)	25 (96.1)	22 (100)	43 (97.7)	44 (97.7)	< 0.001
CIP	–	–	–	2 (100)	–	0 (0)	–	1 (33.3)	–	0.138
LEV	4 (100)	21 (72.4)	42 (84)	17 (89.4)	21 (100)	10 (100)	14 (87.5)	32 (86.4)	37 (94.8)	< 0.001
IMI	22 (81.4)	36 (87.8)	44 (78.5)	18 (81.8)	26 (96.2)	22 (100)	3 (100)	12 (100)	23 (100)	< 0.001
MEM	21 (80.7)	35 (83.3)	46 (80.7)	18 (78.2)	26 (100)	36 (97.2)	26 (100)	38 (95)	2 (100)	< 0.001
GM	13 (86.6)	31 (88.5)	43 (84.3)	17 (80.9)	6 (46.1)	11(42.3)	4 (57.1)	14 (50)	–	< 0.001
AK	11 (100)	29 (85.2)	41 (78.8)	18 (85.7)	15 (71.4)	16 (50)	16 (72.7)	5 (71.4)	0 (0)	< 0.001
PTZ	4 (100)	27 (79.4)	48 (85.7)	18 (90)	23 (95.8)	3 (100)	–	37 (90.2)	39 (97.5)	< 0.001
CPM	4 (80)	32 (91.4)	49 (89)	16(88.8)	8 (100)	20 (86.9)	15 (100)	11 (84.6)	–	< 0.001
CTX	10 (100)	24 (92.3)	39 (86.6)	16 (88.8)	19 (100)	27 (100)	19 (100)	35 (92.1)	44 (100)	< 0.001
CRO	20 (86.9)	24 (92.3)	49 (90.7)	17 (89.4)	25 (100)	29 (96.6)	23 (95.8)	41 (93.1)	13 (100)	< 0.001

SAM (Ampicillin-Sulbactam), CAZ (Ceftazidime), CIP (Ciprofloxacin), LEV (Levofloxacin), IMI (Imipenem), MEM (Meropenem), GM (Gentamicin), AK (Amikacin), PTZ (Piperacillin-Tazobactam), CPM (Cefepime), CTX (Cefotaxime), and CRO (Ceftriaxone).

Variables were described as the frequency (%).

*Samples for which antibiotic resistance testing has not been performed are marked with "–".

CAZ (Ceftazidime), GM (Gentamicin), PTZ (Piperacillin-Tazobactam), AK (Amikacin), CPM (Cefepime), CIP (Ciprofloxacin), LEV (Levofloxacin), IMI (Imipenem), and MEM (Meropenem).

Variables were described as the frequency (%).

*Samples for which antibiotic resistance testing has not been performed are marked with "–".

## Discussion

AMR is a severe global threat that raises growing health system concerns. However, this issue has received insufficient attention during the recent pandemic. The research found a significant decrease in AMR surveillance during the COVID-19 pandemic, which may have limited the ability to provide information on actual AMR changes and raised the possibility of an AMR silent pandemic [10].

In recent years, the global incidence of infections caused by gram-negative bacteria resistant to antibiotics has increased. It has been predicted that up to two million people in the United States will contract an antibiotic-resistant bacterial infection each year, resulting in over 23,000 fatalities [11]. Resistant gram-negative bacteria, such as *E. coli*, *P. aeruginosa*, *K. pneumoniae*, and *A. baumannii*, pose a significant threat to public health and impose an economic burden.

To our knowledge, no similar study exists in databases examining the evolution of AMR during the COVID-19 era. However, few studies have revealed unsatisfactory outcomes. A study evaluating antibiotic resistance in limited clinical samples was conducted during the pandemic. Boorgula et al. analyzed 200 clinical samples from 122 COVID-19 patients with secondary infections from April to May 2021 and identified *K. pneumoniae* as the most prevalent bacteria, followed by *A. baumannii*, where isolates exhibited a 6% rise in carbapenem resistance [12]. Another study in Italy reported that despite the correct use of personal protective equipment (PPE), carbapenem-resistant Enterobacteriaceae acquisition increased from 5% in 2019 to 50% during the pandemic [13].

This study revealed an increase in AMR in clinical samples isolated between January 2020 and January 2022 (COVID-19 era) at the Hasheminezhad hospital in Mashhad, Iran. The rate of antibiotic resistance in these bacteria was lower than in the COVID-19 pandemic. Before the pandemic, only one study evaluated AMR in Mashhad between August 2016 and February 2017. The study only examined *E. coli* antibiotic resistance patterns to drug classes and evidenced 64.7% and 4.4% resistance to cephalosporins and carbapenems, respectively [14]. There were other studies conducted in Iran. Sharahi et al. in Iran demonstrated antibiotic resistance in *E. coli* and *K. pneumoniae* prior to the outbreak of COVID-19 from September 2016 to August 2018 [15]. They examined 165 isolates of E. coli and K. pneumoniae and observed that the prevalence of meropenem- and imipenem-resistant *E. coli* isolates were 19.5% and 10.6%, respectively.

In addition, the frequency of *E. coli*'s resistance to meropenem and imipenem in our study was 33.6% and 42.7%, respectively. In addition, 61.5% and 69.2% of *K. pneumoniae* isolates were resistant to meropenem and imipenem, respectively. In our study, the frequency of *K. pneumonia’*s resistance to meropenem and imipenem was 69.4% and 71.7%, respectively.

Another study conducted by Tarafdar et al. in Iran from May 2018 to the end of July 2019 on 98 clinical isolates, 50 *A. baumannii* isolates and 48 *P. aeruginosa* isolates revealed that antibiotic resistance of *P. aeruginosa* to meropenem and imipenem was 45% and 46%, respectively [16]. Conversely, *P. aeruginosa* resistance to meropenem and imipenem was 91.5% and 91.8%, respectively, in our study. Furthermore, antibiotic resistance in A. baumannii resistance to meropenem and imipenem was both 47%; however, resistance to meropenem and imipenem for *A. baumannii* in our study was 88.9% and 88.4%, respectively.

According to Tables 4, 5, 6, 7, all bacteria experienced an increase in AMR during the first year of the COVID-19 pandemic. Although our results need to be interpreted with caution, because of the small number of isolates tested per species and per antibiotic, we feel this is likely due to the early need to combat the pandemic and the unrestricted use of antibiotics during the first year of the outbreak.

This increased risk of an AMR pandemic is heightened in low- and middle-income countries and may have multiple causes [17]. The overuse of antibiotics is the leading cause of AMR. The disparity between the incidence of bacterial infections and the frequency with which antibiotics are administered suggests that these antibiotics have been overprescribed. Antibiotic overuse in COVID-19 patients can increase the selective pressure for AMR. AMR may be a long-term consequence of the COVID-19 outbreak due to antibiotic overuse, healthcare worker fatigue, and a limited capacity to monitor antibiotic-resistant organisms.

According to a meta-analysis by Langford et al., antibiotic prescriptions were administered in approximately 75% of cases of COVID-19, while bacterial co-infection occurred in less than 10%. [18]. COVID-19 patients receive antibiotics for a variety of reasons. Diagnostics used to differentiate between bacterial and viral infections may be ineffective or time-consuming when rapid treatment is necessary. For example, CRP levels, typically elevated in bacterial infections, may be elevated in COVID-19 cases.

Antibiotics will be administered to a substantial number of hospitalized COVID-19 patients due to the lack of diagnostic test confirmation. COVID-19 patients may develop secondary co-infections requiring antimicrobial therapy; however, as stated previously, the prevalence of bacterial co-infection among COVID-19 patients is lower than the rate of antibiotic use. A patient with COVID-19 may exhibit non-specific symptoms, and this overlap may lead to antibiotic overuse. As the evidence indicates that chloroquine, hydroxychloroquine, and azithromycin are ineffective against COVID-19, their use in various settings has been suspended. However, a lack of knowledge and the absence of alternative therapeutic options have contributed to the continued use of these drugs in various circumstances [19].

Most healthcare workers were required to respond to the COVID-19 outbreak, limiting their availability for AMR measures. Self-medication with antibiotics is increasing, and obtaining professional guidance prior to prescribing antimicrobials is becoming increasingly difficult. Furthermore, increased use of sanitizers and other biocidal chemicals, along with their environmental exposure, and challenges adhering to conventional infection prevention and control precautions for health personnel due to extended shifts using the same PPE and possible equipment shortages are prevalent. During the COVID-19 outbreak, significant funds were allocated to lab equipment and patient care, while AMR evaluation received little support from associated organizations. Experts have issued warnings concerning AMR in the COVID-19 era for various reasons, including the longer hospital stays for COVID-19 patients [4, 20].

## Conclusion

Gram-negative bacteria are one of the leading causes of significant antibiotic resistance during the COVID-19 pandemic (2020–2022) in clinical settings. Antibiotic resistance is increasing due to various factors, most notably antibiotic overuse. The development and spread of genes for antibiotic resistance in bacteria will further limit treatment options and jeopardize global public health. This study suggests that *E. coli*, *P. aeruginosa*, *K. pneumoniae*, and *A. baumannii* strains exhibited a pattern of increasing antibiotic resistance during the COVID-19 pandemic, particularly during the first year.

## Data Availability

The data that support the findings of this study are available from Mums but restrictions apply to the availability of these data, which were used under license for the current study, and so are not publicly available. Data are however available from the authors upon reasonable request and with permission of MUMS.
